# Sit-to-stand and stand-to-sit transitions stimulate cardiac autonomic regulation and lead to age-dependent short-term patterns in heart rate

**DOI:** 10.3389/fphys.2026.1733672

**Published:** 2026-02-19

**Authors:** Max J. Heidelbach, Silke Lange, Friedrich Edelhäuser, Dirk Cysarz

**Affiliations:** 1 Integrated Curriculum for Anthroposophic Medicine, University of Witten/Herdecke, Witten, Germany; 2 Interprofessional Graduate School in Integrative Medicine and Health (IGIM), University of Witten/Herdecke, Witten, Germany

**Keywords:** heart rate, heart rate variability, physical activity, sedentary behavior, sitting down, standing up, transient oscillation, blood pressure

## Abstract

**Background:**

Sedentary behaviors pose health risks, which can be mitigated by regular exercise. The impact of everyday movements on cardiac autonomic regulation is not well-established. The present study investigated whether standing up and sitting down from a chair leads to a reproducible transition pattern in heart rate. Furthermore, age-related changes in this pattern were assessed.

**Methods:**

Forty healthy participants (18–65 years, 22 female) performed eight repetitions of standing up from a chair and sitting down. Electrocardiogram and accelerometer data were recorded throughout the procedure. The average RR interval series was used to quantify the transition pattern evoked by the movement.

**Results:**

Both movements resulted in a transition pattern in the RR interval and corresponding heart rate (HR) series within 30 s. After the initiation of movement, a local maximum HR appeared at a median of 8.1 s (standing up, HR_stand max_ = 78.4 bpm vs. HR_sit_ = 62.4 bpm) and 2.1 s (sitting down, HR_sit max_ = 78.0 bpm vs. HR_stand_ = 70.2 bpm), followed by a local minimum HR at a median of 18.0 s (standing up, HR_stand min_ = 68.0 bpm; *p* < 0.001 vs. HR_stand max_ and HR_sit_) and 14.1 s (sitting down, HR_sit-min_ = 60.7 bpm; *p* < 0.001 vs. HR_sit max_ and HR_stand_). Subsequently, the HR increased until a steady state was reached. The time to reach the local minimum increased with age for both movements. Furthermore, the standing up HR_min_/HR_max_ decreased with increasing age.

**Conclusion:**

Standing up from a chair and sitting down each briefly stimulate cardiac autonomic regulation. The transition process partially slows down and becomes less pronounced with increasing age. Increasing the daily amount of standing up contributes to cardiac autonomic regulation flexibility and helps mitigating cardiovascular risks from sedentary behaviors.

**Trial Registration:**

https://drks.de, Unique identifier: DRKS00021712.

## Introduction

Sedentary behaviors - waking behaviors with low energy expenditure that occur in a sitting, reclined, or lying position (Tremblay et al., 2017) - are ubiquitous in modern societies, and further increased during the coronavirus disease 2019 (COVID-19) pandemic due to lockdowns and other social restrictions (Runacres et al., 2021). Such behaviors also increase with advancing age, and are particularly common in older individuals (>70 years) (Asher et al., 2025). Sedentary time is associated with negative consequences for cardiovascular health; the more time a person spends sedentary, the higher their risk for cardiovascular disease (Pandey et al., 2016; Jingjie et al., 2022). In contrast to sedentary behaviors, physical activity is associated with a reduction in cardiovascular and all-cause mortality (Nocon et al., 2008; Wahid et al., 2016).

Light-intensity physical activities are part of daily life; for example, standing up from a chair, walking a few steps, and sitting down again. Adults perform these movements approximately 50 times per day (Bohannon, 2015; Smith et al., 2015). However, these physical activities are rarely considered relevant exercise because the energy expenditure is low and the active duration is short (Herrmann et al., 2024). Moderate to vigorous physical activity is often recommended to improve cardiovascular health (Bull et al., 2020). Thus, it is surprising that increasing only the frequency of standing up from 45 to 70 times a day for 3 months has been found to decrease blood pressure in postmenopausal women (Hartman et al., 2025).

The impact of everyday movements, specifically light-intensity physical activity, on immediate cardiac autonomic regulation has not often been addressed. Recently, we have demonstrated that everyday movements, such as kneeling on one knee (“tie your shoes”) or raising the arms (“expressive yawning”), trigger a pronounced transient response (approximately 30 s) in cardiac autonomic regulation (Heidelbach et al., 2024).

In this study, we investigated transient regulatory responses in the duration between successive heartbeats (RR interval series) following a basic everyday movement to interrupt sedentary behavior: standing up from a chair and sitting down again. To our knowledge, cardiovascular responses to standing up have been investigated during the transition from the supine to the standing position, addressing the impact of the orthostatic challenge on the autonomic nervous system (Ewing et al., 1978; Ewing et al., 1980; Borst et al., 1982). Here, we investigated whether standing up from a chair and sitting down elicits a marked, reproducible response in cardiac autonomic regulation, as reflected in the pattern of the RR interval series. Furthermore, we addressed age-related changes in this regulatory response, as it is known that cardiac autonomic regulation weakens with age. This is evidenced by, for example, age-related decreases in heart rate variability (HRV) (Umetani et al., 1998; Pikkujamsa et al., 1999; Almeida-Santos et al., 2016). We demonstrate that the regulatory responses in the RR interval series after standing up and sitting down also partly depend on age.

## Methods

### Participants

Forty healthy adults (aged 18–65 years, 22 women) participated in the study. All participants were in good health. The body mass index (BMI) of most of the participants ranged from 18.1 to 25.5 kg/m^2^, indicating a normal BMI. Two participants aged >57 years had a slightly higher BMI (>25 kg/m^2^) and were included to consider age-related weight gain (Haftenberger et al., 2016). Participants undergoing permanent medication treatment (with the exception of oral contraceptives) and those with cardiovascular diseases (hypertension, hypotension, or heart failure), diabetes mellitus, thyroid gland diseases, back, knee, or hip pain, any other movement-limiting disease, or acute infection (including fever), were excluded.

### Experimental protocol

On eight different study days, groups of five participants each performed a sequence of standing up from a chair and sitting down. We aimed to attain movements as similar as possible in all participants. Hence, all participants synchronized their movements with a video that was shown in front of the participants and the study representative simultaneously demonstrated the movement. The sequence began with 2 min of sitting quietly in a chair to acclimate the cardiovascular system to the sitting position. The participants then stood up from the chair at a normal pace and stood still for the following 30 s. Next, the participants sat down at a normal pace and remained seated for 30 s. This procedure was repeated eight times; thus, each participant stood up from the chair eight times and sat down eight times. The entire protocol lasted a total of 10 minutes. The individuals were instructed to perform the procedure silently, and breathing was not restricted.

### Measurements

The participants were equipped with a 1-channel echocardiogram (ECG) recording device (Faros 180, Bittium Corp., Finland) on the chest (modified lead II). The ECG was sampled at 250 Hz. The device simultaneously recorded movements with a 3D accelerometer. The movement signal was sampled at 100 Hz per axis (x, y, and z).

The device automatically detected the times of the R-peak in the ECG. The recordings were subsequently checked for artefacts and manually corrected if necessary (<0.1% of all R-peaks). The times of R-peaks were used to calculate the RR tachogram, the series of times between successive R-peaks. The RR tachogram was resampled at 4 Hz to form a proper RR time series. The instantaneous heart rate (HR) was calculated by the relationship HR = 60/RR. The RR time series was used for further calculations.

### Analysis of patterns in the RR interval series

**FIGURE 1:**
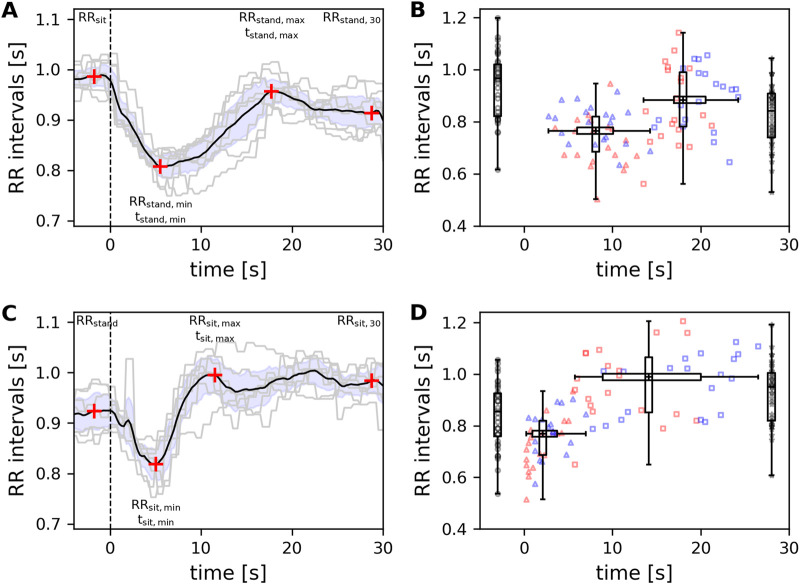
Examples of RR intervals during **(A)** standing up and **(C)** sitting down. The grey lines show the RR intervals during the eight repetitions, the black line represents the average RR interval series, and the blue area represents the confidence interval of the average series. The vertical dashed line indicates the start of standing up and sitting down, respectively. Red crosses specify the four RR intervals used to quantify the transition pattern. **(A)** RR intervals during standing up were quantified 2 s before the movement starts, RR_sit_; at the time t_stand,min_ of the minimum RR interval, RR_stand,min_; at the time t_stand,max_ of the maximum RR interval, RR_stand,max_; and after 30 s, RR_stand,30s_. **(C)** RR intervals during sitting down were quantified analogously and resulted in RR_stand_, RR_sit, min_at time t_sit,min_, RR_sit,max_ at time t_sit,max_ and RR_sit,30_. **(B)** RR_sit_ (symbol 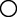), RR_stand,min_ (symbol 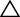), RR_stand,max_ (symbol 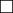), and RR_stand,30_ (symbol 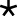) of all 40 subjects during standing up. The four vertical boxplots indicate the distribution of the four RR intervals; the horizontal boxplots indicate the distribution of the times t_stand,min_ and t_stand,max_. **(D)** RR_stand_ (symbol 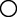), RR_sit,min_ (symbol 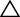), RR_sit,max_ (symbol 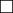), and RR_sit,30_ (symbol 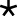) of all 40 subjects during sitting down. The vertical boxplots indicate the distribution of the four RR intervals; the horizontal boxplots indicate the distribution of the times t_sit,min_ and t_sit,max_. Red symbols: age ≤40 years, blue symbols: age >40 years.

Standing up from and sitting down in the chair were analyzed separately. The initiation of both movements resulted in clear, movement-related changes in the accelerometer signal (Janssen et al., 2008). The precise timings of the changes in the accelerometer signal were detected by a changepoint algorithm. The times of the changepoints were used to form 30-s segments in the RR interval series. As the analysis focused on transient behavior in the RR interval series, the start of the segment was set to 2 s before the onset of the movement to include preceding cardiac autonomic regulation. The average RR time series was calculated across all eight repetitions of standing up or sitting down (see examples in Figures 1A,C).

Each separate RR time series and the average RR time series showed a clear transition pattern for standing up and sitting down in all participants. During standing up, RR intervals decreased by approximately 5–8 s to reach a local minimum (see Figure 1A, black line). Subsequently, RR intervals increased to reach a local maximum that appeared approximately 15–20 s after the start of the movement. At the end of the 30-s epoch, the RR intervals slightly increased again. Hence, the average RR interval was quantified at four different times (see Figure 1A, red crosses): 2 s before standing up started (i.e., in the sitting position, RR_sit_), during the local minimum after standing up (RR_stand,min_), during the local maximum (RR_stand,max_), and at the end of the 30 s epoch (RR_stand,30_). Furthermore, changes of RR_stand,min_, RR_stand,max_ and RR_stand,30_ relative to RR_sit_ were calculated. The times of the local minimum (t_stand,min_) and maximum (t_stand,max_) were also obtained. The times had a resolution of 0.25 s, according to the resampling rate of the RR interval time series.

The transition pattern in the RR interval series during sitting movements was analogous to that while standing up. After sitting down, a local minimum appeared after approximately 2-5 s (see Figure 1C, black line). Subsequently, the RR intervals increased to reach a local maximum, approximately 10–20 s after the start of the movement. At the end of the 30-s epoch, the RR intervals slightly increased again. Consistent with the standing-up measurement, the average RR interval was quantified at four different times (see Figure 1C, red crosses): 2 s before sitting down (i.e., in the standing position, RR_stand_), during the local minimum after sitting down (RR_sit,min_), during the local maximum (RR_sit,max_), and at the end of the 30-s epoch (RR_sit,30_). Furthermore, changes of RR_sit,min_, RR_sit,max_ and RR_sit,30_ relative to RR_stand_ were calculated. The times of the local minimum (t_sit,min_) and maximum (t_sit,max_) were also obtained.

### Statistical calculations

The aim was to quantify the dynamics of the transient pattern in the RR interval series while standing up and sitting down, as well as its dependency on age. Nonparametric statistical procedures were employed because the sample size was relatively small; in addition, some parameters did not exhibit a normal distribution, as indicated by a low *p-*value in the Shapiro–Wilk test for normality. Accordingly, the median and the interquartile range (1st and 3rd quartiles) were used as descriptors of the parameter distributions. Differences between the four RR intervals during standing up and sitting down were assessed using the nonparametric Friedman test for related samples. In case of significant differences, the Conover test was used for pairwise comparisons. This approach included adjustments for multiple comparisons (Conover, 1999). Furthermore, we calculated the non-parametric effect size Cliff’s Delta between the four RR intervals. We note that parametric procedures (e.g., Cohen d effect size) show similar effect sizes (Cliff, 1996). Analogous to physiological studies that investigated standing up from the supine position, we also calculated the RR_max_/RR_min_ as an index of the relative amplitude of the transient oscillation in the RR interval series. This quotient is equivalent to the 30:15 HR ratio (the maximum RR interval at approximately 30 heartbeats divided by the minimum RR interval at 15 heartbeats after standing up), which is used in many studies to quantify the transient oscillation in the RR interval series after standing from the supine position (Ten Harkel et al., 1990).

The differences in the times from the start of the movement to the local minimum and maximum were assessed between standing up and sitting down using the paired Wilcoxon signed-rank test.

Age-related changes in the RR intervals, particularly those of the local minimum and maximum during standing up and sitting down, were analyzed by plotting the respective RR intervals against the participants’ age. Pearson’s correlation coefficient r was calculated to quantify a linear dependency of the minimum and maximum RR interval on age (r_age_). Furthermore, the times to reach the minimum and maximum in the RR interval, t_min_ and t_max_, were assessed with respect to age-related changes. BMI may also depend on age (Haftenberger et al., 2016); thus, BMI-related changes in the parameters were also analyzed (r_BMI_). The slope and intercept of the linear regression line were calculated in cases of a significant correlation.

To control for the influence of BMI and age, partial correlation coefficients r_p,age_ and r_p,bmi_ were calculated. These represented the dependency of the parameters on age, controlling for BMI (r_p,age_), and the dependency of the parameters on BMI, controlling for age (r_p,bmi_).

A *p-*value <0.05 was considered statistically significant. The analyses and all statistical procedures were conducted using custom Julia and R scripts.

### Trial registration and ethics

The study procedures were performed in accordance with the tenets of the Declaration of Helsinki. The trial was registered with the German Clinical Trial Register (DRKS-ID: DRKS 00021712) and the World Health Organization International Clinical Trials Registry Platform (ICTRP). Informed consent was obtained from all participants. The protocol was approved by the institutional ethics review board of the University of Witten/Herdecke on 28/07/2020 (No: 90/2020).

## Results

### Transient patterns in the RR interval series while standing up and sitting down

The RR interval series showed one oscillation—a transient pattern—after standing up and sitting down. This pattern was replicable within (see examples in Figures 1A,C) and between (see Figures 1B,D) participants.

The results related to standing up from the chair are presented in Table 1 and Figure 1B. The median RR interval during sitting, RR_sit_, was 967 ms (822–1020 ms; 62.4 bpm; 58.8–73.0 bpm) and decreased by 20.1% to 765 ms (684–820 ms; 78.4 bpm; 73.2–87.8 bpm) during the minimum after standing up (RR_stand,min_). The local minimum appeared 8.1 s (6.0–10.1 s) after the start of standing up (t_stand,min_). Next, the RR intervals increased to a local maximum (RR_stand,max_: 883 ms; 704–908 ms; 68.0 bpm; 66.1–85.2 bpm). Nevertheless, this represents a decrease of 6.8% compared to RR_sit_. The local maximum appeared 18.0 s (16.9–20.6 s) after the start of standing up (t_stand,max_). At the end of the 30-s period, the median RR interval during standing, RR_stand,30s_, was 841 ms (704–907 ms; 71.3 bpm; 66.2–85.2 bpm), a decrease of 11.9% compared to RR_sit_. Significant differences were observed between the RR intervals at the four time points (*p* < 0.001). All pairwise comparisons between the RR intervals at the four time points showed significant differences (*p* < 0.001). Cliff Delta indicated a large effect size in RR_sit_ vs. RR_stand,min_ (0.76), RR_sit_ vs. RR_stand,30s_ (0.53), RR_stand,min_ vs. RR_stand,max_ (−0.53). A medium effect size was observed for RR_stand,min_ vs. RR_stand,30s_ (−0.35) and a small effect size was observed for RR_sit_ vs. RR_stand,max_ (0.29) and RR_stand,max_ vs. RR_stand,30s_ (0.26). The median ratio RR_stand,max_/RR_stand,min_ (or the equivalent, HR_stand min_/HR_stand max_) was 1.15 (1.10–1.22), indicating that the maximum RR interval was 15% higher than the minimum RR interval.

**TABLE 1:** Transient pattern quantification of the RR interval series while standing up and sitting down. The patterns are quantified by four successive instances: (1) the initial value during sitting and standing, (2) the minimum in the RR intervals after the start of the movement, (3) the maximum in the RR intervals, and (4) the RR intervals after 30 s. The ratio RR_max_/RR_min_ quantifies the relative amplitude of the transition oscillation.

Standing up	RR_sit_ [ms]	RR_stand,min_ [ms]	RR_stand,max_ [ms]	RR_stand,30s_ [ms]	RR_stand,max_/RR_stand,min_
Median*	967	765	883	841	1.15^#^
IQR	822–1020	684–820	780–989	740–908	1.10–1.22
Rel. change	1	0.799	0.932	0.881	
		t_stand min_ [s]	t_stand max_ [s]		
Median		8.1^#^	18.0^#^		
IQR		6.0–10.1	16.9–20.6		
Sitting down					
Median*	855	769	989	952	1.28
IQR	759–926	687–820	851–1065	820–1004	1.21–1.32
Rel. change	1	0.903	1.126	1.094	
		t_sit min_ [s]	t_sit max_ [s]		
Median		2.1	14.1		
IQR		0.9–3.8	8.9–20.0		

* p_Friedman_ < 0.001, the four RRs, differ from each other in both movements (*p* < 0.001).

#*p* < 0.001 vs. the respective value during sitting down (t_sit min_, t_sit max_, and RR_max_/RR_min_).

Sitting down showed a qualitatively similar pattern in the RR interval series to that observed with standing up (Figure 1D). The median RR interval during standing at the beginning, RR_stand_, was 855 ms (759–926 ms; 70.2 bpm; 64.8–79.1 bpm). The local minimum appeared 2.1 s (0.9–3.8 s) after sitting down (t_sit,min_), and the median RR interval RR_sit,min_ decreased by 9.7% to 769 ms (687–820 ms; 78.0 bpm; 73.2–87.3 bpm). This local minimum was similar to that observed during standing up. The local maximum occurred 14.1 s (8.9–20.0 s) after sitting down (t_sit,max_), and the median RR interval RR_sit,max_ increased to 989 ms (851–1065 ms; 60.7 bpm; 56.3–70.5 bpm). This represents an increase of 12.6% compared to RR_stand_. This local maximum was higher than RR_stand,max_ during standing up (*p* < 0.001). At the end of the 30-s period, the median RR interval during sitting, RR_sit,30s_, was 952 ms (820–1004 ms; 63.0 bpm; 59.8–73.2 bpm), an increase of 9.4% compared to RR_stand_. Again, the Friedman test indicated significant differences across the RR intervals at the four time points *(p <* 0.001), and all pairwise comparisons indicated significant differences (*p* < 0.001). Cliff Delta indicated a large effect size in RR_stand_ vs. RR_sit,max_ (−0.50), RR_sit,min_ vs. RR_sit,max_ (−0.82) and RR_sit,min_ vs. RR_sit,30s_ (−0.75). A medium effect size was observed for RR_stand_ vs. RR_sit,min_ (−0.42) and RR_stand_ vs. RR_sit,30_ (−0.40). A small effect size was observed for RR_sit,max_ vs. RR_sit,30_ (0.20). The median ratio RR_sit,max_/RR_sit,min_ (or, equivalently HR_sit min_/HR_sit max_) was 1.28 (1.21–1.32), indicating that the maximum RR interval was 28% higher than the minimum RR interval. This ratio was higher than that during standing up (*p* < 0.001).

The median RR interval during standing after the movement of standing up, RR_stand,30s_, and during standing at the beginning of sitting down, RR_stand_, were significantly different (841 vs. 855 ms; 71.3 vs. 70.2 bpm; *p* < 0.001). Furthermore, the median RR interval during sitting after sitting down, RR_sit,30s_, and during sitting just before standing up, RR_sit_, were also different (952 vs. 967 ms; 63.0 vs. 62.0 bpm; *p* < 0.001). These small but significant differences suggest that the 30-s period was insufficient for cardiovascular regulation to fully adapt to the new condition after standing up or sitting down.

### Age and BMI-related changes

**FIGURE 2:**
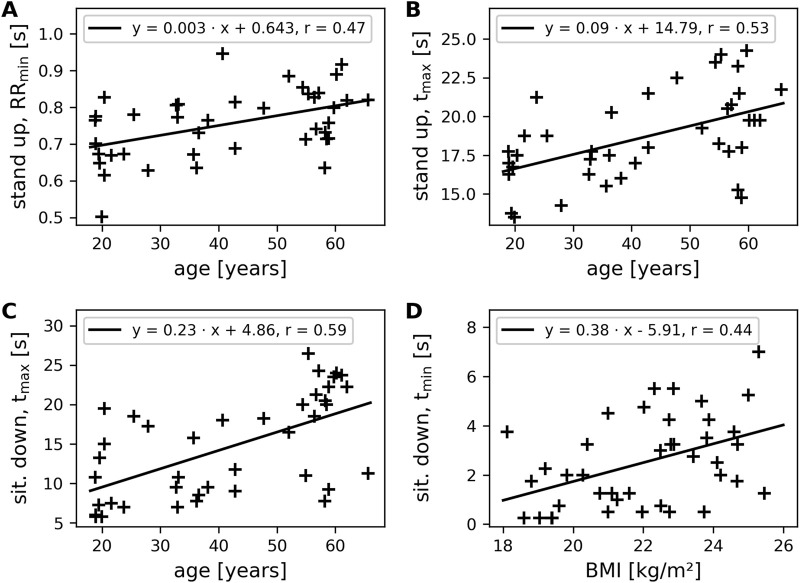
The four parameters most strongly correlated with age or BMI. **(A)** RR_stand,min_ after standing up, **(B)** time t_stand,max_ to reach RR_stand,max_ after standing up, and **(C)** time t_sit,max_ to reach RR_sit,max_ after sitting down show age-related changes. **(D)** Time t_sit_,_min_ to reach RR_sit,min_ after sitting down shows BMI-related changes. The legend in each diagram displays the parameters of the linear regression line and the correlation coefficient. The slope indicates the increase per year **(A–C)** or the increase per kg/m^2^ **(D)**.

Notably, BMI showed age-related changes (r_age_ = 0.44, *p* < 0.01); according to the linear regression, the average BMI increased 0.06 kg/m^2^ per year. For standing up, the minimum RR interval during the movement, RR_stand,min_ increased with increasing age (r_age_ = 0.47, *p* < 0.01; average increase: 3 ms per year; see Table 2; Figure 2A). Controlling for BMI in these age-related changes still yielded a significant correlation (r_part,age_ = 0.35, *p* < 0.05). The time to reach the maximum RR interval, t_stand,max_, increased with advancing age (r_age_ = 0.53, *p* < 0.001; average increase: 0.1 s per year; see Figure 2B) even after controlling for BMI (r_part,age_ = 0.52, *p* < 0.001). The ratio RR_stand,max_/RR_stand,min_ decreased with increasing age (r_age_ = −0.50, *p* < 0.001; average decrease: −0.003 per year), and this decrease remained after controlling for BMI (r_part,age_ = −0.56, *p* < 0.001). BMI-related changes were observed for the maximum RR interval, RR_stand,max_, (r_BMI_ = 0.34, *p* < 0.05; average increase: 22 ms per kg/m^2^) and persisted after controlling for age (r_part,BMI_ = 0.32, *p* < 0.05). Furthermore, the RR interval after 30 s, RR_stand,30s_, showed BMI-related changes (r_BMI_ = 0.34, *p* < 0.05; average increase: 19 ms per kg/m^2^). However, these BMI-related changes disappeared when controlling for age.

**TABLE 2:** Age- and BMI-related changes during standing up and sitting down per parameters quantified by Pearson’s correlation coefficient r and partial correlation coefficient r_part_ to control for BMI or age.

Standing up	r_age_	r_part,age_, (contr for BMI)	r_BMI_	r_part,BMI_, (contr for age)
RR_sit_	0.10	0.02	0.17	0.15
RR_stand,min_	0.47 **	0.35*	0.27	0.18
RR_stand,max_	0.13	−0.03	0.34*	0.32*
RR_stand,30s_	0.24	0.11	0.34*	0.27
t_stand,min_	−0.06	−0.03	−0.09	−0.07
t_stand,max_	0.53***	0.52***	0.15	−0.11
RR_max_/RR_min_	−0.50***	−0.56***	0.00	0.28
Sitting down				
RR_stand_	0.20	0.06	0.34*	0.29
RR_sit,min_	0.32*	0.19	0.36*	0.26
RR_sit,max_	0.06	−0.02	0.17	0.16
RR_sit,30s_	0.14	0.07	0.18	0.14
t_sit,min_	0.41**	0.27	0.44 **	0.32*
t_sit,max_	0.59***	0.51 ***	0.38*	0.17
RR_max_/RR_min_	−0.38*	−0.31	−0.27	−0.12

*p < 0.05, **p < 0.01, ***p < 0.001.

Sitting down also showed age- and BMI-related changes. The minimum RR interval, RR_sit,min_. increased with increasing age (r_age_ = 0.32, *p* < 0.05; average increase: 2 ms per year). However, these age-related changes disappeared when controlling for BMI (r_p,age_ = 0.19). The time to reach the minimum RR interval, t_sit,min_, showed age-related changes (r_age_ = 0.41, *p* < 0.01; average increase 0.05 s per year). However, when controlling for BMI, this correlation disappeared. The time to reach the maximum RR interval, t_sit,max_, showed the strongest age-related changes (r_age_ = 0.59, *p* < 0.001; average increase 0.23 s per year; see Figure 2C), which remained significant after controlling for BMI (r_p,age_ = 0.51, *p* < 0.001). The ratio RR_sit,max_/RR_sit,min_ decreased with increasing age (r_age_ = −0.38, *p* < 0.05; average decrease: −0.003 per year). This decrease disappeared when controlling for age. BMI-related changes were observed for the RR interval during standing before sitting down, RR_stand_, (r_BMI_ = 0.34, *p* < 0.05; average increase 20 ms per kg/m^2^). However, this correlation disappeared after controlling for age. The minimum RR interval, RR_sit,min_, also showed BMI-related changes (r_BMI_ = 0.36, *p* < 0.05; average increase 16 ms per kg/m^2^). This correlation also disappeared after controlling for age. The time to reach the minimum RR interval, t_sit,min_, showed BMI-related changes (r_BMI_ = 0.44, *p* < 0.01; average increase 0.38 s per kg/m^2^; see Figure 2D) that persisted after controlling for age (r_p,BMI_ = 0.32, *p* < 0.05). The time to reach the maximum RR interval, t_sit,max_, showed BMI-related changes (r_BMI_ = 0.38, *p* < 0.05; average increase 1.2 s per kg/m^2^), but these were not present after controlling for age.

## Discussion

Light physical activity, such as standing up from a chair and sitting down, is rarely considered relevant for cardiovascular risk reduction because of the short duration and low energy expenditure of such movements (Kralj et al., 1990; Ashford and De Souza, 2000; Herrmann et al., 2024). Here, we showed that standing up from a chair leads to a regulatory response that can be described as a transition oscillation - a specific pattern in the RR interval series of approximately 30 s - despite the movement itself only taking approximately 3 s. After standing up the RR intervals decrease by approx. 20% followed by an increase to approx. 93% of the initial RR interval. Finally, after 30 s of standing the RR intervals decrease by 12% compared to sitting. Sitting down, while also taking only a short amount of time, shows a transition pattern with a faster adaptation response in cardiac autonomic regulation (approximately 4 s faster). After sitting down the RR intervals counter-intuitively decrease by approx. 10% followed by an increase of approx. 12% compared to standing. Finally, after 30 s of sitting the RR interval increase by almost 10% compared to standing. During both, standing up and sitting down, especially the large transitions show a large effect size indicating relevant changes in cardiac autonomic control. The adaptation dynamics of standing up and sitting down slow down with increasing age. The time to reach the maximum and minimum RR intervals after standing up or sitting down increases with age, with the HR increase after standing up or sitting down slowing down. The relative amplitude also decreases with increasing age.

In terms of RR intervals after standing up from a chair, our findings were similar to reports on standing up from the supine position. Standing up from the supine position has been studied in detail in the context of orthostatic (mal)adaptation, and is associated with a marked transition oscillation in the blood pressure and RR intervals of healthy individuals (Ewing et al., 1978; Ewing et al., 1980; Borst et al., 1982; Borst et al., 1984; Harms et al., 2021). These changes occur in the first 30 s of active standing (Ewing et al., 1980; Borst et al., 1982; Harms et al., 2021). Standing up from the supine position takes approximately 3 s (Sprangers et al., 1991). This brief effort leads to an immediate decrease in RR intervals, caused by an increase in HR and a slight increase in diastolic blood pressure, with systolic blood pressure unaffected (Harms et al., 2021). Simultaneously, as blood follows gravity, the central blood volume decreases and shifts towards the lower parts of the body, especially the legs. This is accompanied by a decrease in total peripheral resistance (Sprangers et al., 1991). Consequently, systolic and diastolic blood pressure decrease rapidly. As a quick cardiovascular countermeasure, to stabilize blood pressure and avoid fainting caused by too low blood pressure in the brain, RR intervals decrease as HR increases (Wieling et al., 2009). The continuous contractions of the leg muscles during standing lead to increased oxygen consumption, increased venous return, and a subsequent increase in cardiac output (Boushel, 2010; Calbet et al., 2015). Total peripheral resistance also increases across the time course (Sprangers et al., 1991). These effects stabilize blood pressure; thus, after the initial drop in the RR interval, it increases again as HR decreases.

These cardiovascular adaptation mechanisms also apply to standing up from a chair. The movement speed of standing up from a chair is similar to that of standing up from the supine position; it is a brief effort, taking approximately 3 s (Kralj et al., 1990; Ashford and De Souza, 2000). The transition oscillation in blood pressure in the transition from sitting to standing is slightly less pronounced than the transition from the supine position to standing (Ten Harkel et al., 1990; Harms et al., 2021). Nevertheless, a decrease in total peripheral resistance should also be observable after standing up from the sitting position, analogous to the decrease in total peripheral resistance after standing up from the supine position (Sprangers et al., 1991). It appears that together, this decrease in total peripheral resistance and the simultaneous blood shift towards the legs caused by gravity lead to the decrease in blood pressure. This decrease is then counterbalanced by a decrease in RR intervals through an increase in HR, until blood pressure is stabilized.

Sitting down on a chair also produced a transition oscillation in the RR interval series that has not previously been reported. The movement of sitting down also takes approximately 2–3 s; hence, the speed and effort are comparable to those when standing up (Kralj et al., 1990; Ashford and De Souza, 2000). Despite this, the oscillation in the RR interval series was faster than when standing up, i.e., the times t_sit,min_ is lower than t_stand,min_ and t_sit,max_ is lower than t_stand,max_. The decrease in RR intervals - from the increase in HR - during and after sitting down appears to be caused by the contraction of several muscles that facilitate the movement, such as the hip, gluteal, abdominal, and back muscles (Ashford and De Souza, 2000). Similar to standing up, the contraction of these muscles leads to an increase in venous return and a subsequent increase in cardiac output (Boushel, 2010; Calbet et al., 2015). This increase causes an increase in HR and thus a decrease in the RR intervals. As sitting down only takes a few seconds, many of the involved muscles quickly relax (Ashford and De Souza, 2000). Hence, venous return decreases again, and the adaptation process finishes. Blood pressure has been modeled during the transition from sitting to standing using, e.g., nonlinear features of pressure regulation (Olufsen et al., 2005). Such models could be used to extract relevant parameters that reflect changes in the cardiac autonomic regulation during standing up and sitting down. These parameters may help to differentiate between inter-individual variability from essential regulatory features.

Cardiac autonomic regulation changes with age, with autonomic control decreasing with increasing age (Abhishekh et al., 2013). However, the time needed for the first response of cardiac autonomic regulation after movement initiation does not change with age: the times taken for t_stand,min_ and t_sit,min_ to reach the minimum RR interval (maximum HR) after standing up or sitting down are constant indicating that the maintenance of blood pressure after the movement initiation is essential regardless of age. The next aspect of regulation appears to be less essential, because the times for t_stand,max_ and t_sit,max_ to reach the maximum RR interval and minimum HR, in both standing up and sitting down, increase with advancing age. Furthermore, the relative amplitude RR_stand max_/RR_stand min_ after standing up also decreased with increasing age, consistent with previous findings (Ewing et al., 1978; Vita et al., 1986; Agelink et al., 2001).

The BMI-related changes were strongest for the time t_min,sit_ to reach the minimum RR interval and maximum HR after sitting down. The higher the BMI, the longer it took to reach the minimum RR interval. The regulation of muscle blood flow is altered in obese individuals (Hodnett and Hester, 2007; Stapleton et al., 2008) and, hence, appears to change with increasing weight. BMI-related changes were also observed in the maximum RR interval RR_stand,max_ after standing up. The higher the BMI, the higher the maximum RR interval (minimum HR) was after standing up. Again, an altered muscle blood flow could be responsible for this relationship (Hodnett and Hester, 2007; Stapleton et al., 2008). Other aspects of the transition oscillation during standing up did not show BMI-associated alterations.

Each transition oscillation in the RR interval, i.e., HR, series during standing up and sitting down indicates a short stimulation of cardiac autonomic regulation. Steady-state regulation during sedentary behavior is interrupted to accommodate these required adaptations. If, for example, a person is standing at a desk without moving, the steady-state regulation during standing is interrupted by a brief transition when they sit down. Such transitions appear crucial, with an increase in daily sit-to-stand transitions (from approximately 45–75) alone has been found to decrease blood pressure in postmenopausal women; in contrast, extending the standing time did not influence blood pressure (Hartman et al., 2025). I.e., the increase of the amount of short stimulations of cardiac autonomic regulation during each standing up had a positive impact on blood pressure. Investigations into reducing the frequency of sedentary behavior by increasing the time spent standing, for example, at a desk, have revealed that prolonged standing results in lower leg edema, muscle fatigue, (Wall et al., 2020) and increased arterial stiffness (Caldwell et al., 2018). Thus, replacing one static behavior (sitting) with a different static behavior (standing) did not improve health but rather led to different health risks. To reduce the risks associated with sedentary behavior, half an hour of moderate to vigorous physical activity per day has been recommended (Ekelund et al., 2020). Short-term challenges in cardiac autonomic regulation - caused by standing up from a chair or other everyday movements, such as kneeling to tie shoes (Heidelbach et al., 2024) – have received little attention in this context but also appear to be beneficial, because they stimulate various aspects of autonomic control each time they are performed. Such simple everyday activity may be easier implemented especially in older individuals and patients than moderate to vigorous physical activity (Dogra et al., 2022). This hypothesis could be tested by studying simple interventions that substantially alter the daily frequency of sit-to-stand transitions (e.g., approximately 50 transitions as the average (Bohannon, 2015; Smith et al., 2015) vs. 75 transitions). Generally, a dose-response association between the frequency of activity and a reduction in health risks has been assumed (Franklin et al., 2022; Banach et al., 2023). The present results suggest that everyday movements also contribute to cardiovascular health - even light-intensity physical activity is favorable over sedentary behavior (Del Pozo Cruz et al., 2021; Franklin et al., 2022; Blodgett et al., 2024). This relationship could be tested, e.g., in patients with coronary artery disease. It has been shown that leisure time physical activity reduces the risk for sudden cardiac deaths compared to inactive patients (Tulppo et al., 2020). It could be examined whether increasing the amount of sit-to-stand transitions in these patients, compared to inactivity, also has a positive impact on health outcomes. Furthermore, approaches to analyze the dynamics of the RR intervals using symbolic analysis (Cysarz et al., 2015) could reveal further aspects of short-term cardiac autonomic regulation, as this approach can be applied to short RR interval sequences.

This study has several limitations. The transition oscillation - the pattern formation in the RR interval series during standing up and sitting down - has been demonstrated for RR intervals but still needs to be elucidated in detail, for example, in terms of elements like blood pressure. The simultaneous non-invasive measurement of blood pressure as conducted in studies investigating standing up from the supine position, would enable a deeper insight into vascular regulation. This would help us gain insight into the specific positive cardiovascular effects of these everyday movements and may provide hints about potential negative effects. The age- and BMI-related changes need to be elucidated in more detail because correlation analysis does not imply causality. Increasing the number of participants and including those with higher BMIs would enable to test whether the weaker age- and BMI-related associations of the parameters become stronger with an increased sample size. Furthermore, studies which use movement interventions that focus on age-related or BMI-related changes of cardiac autonomic regulation in everyday activities could reveal the causal role of age and BMI on these regulations.

In conclusion, standing up from a chair and sitting down both lead to marked transition patterns in the RR interval series during and shortly after the movement. The transition pattern becomes partly slower and partly less pronounced with increasing age. Regardless of age, the transition patterns indicate that these movements stimulate cardiac autonomic regulation. The more often standing up and sitting down are performed, the more such regulations are required. Hence, these movements may contribute to cardiovascular health because the involved regulatory mechanisms are stimulated each time, despite being short in duration and low in energy expenditure. The movements are easier to perform than, for example, moderate physical activities; thus, they can be offered to patients with limited mobility. These cardiovascular responses to everyday movements could also serve as an indicator of the regulatory capacity of cardiac autonomic function.

## Data Availability

The raw data supporting the conclusions of this article will be made available by the authors, without undue reservation.
